# Association between SLCO1B1 −521T>C and −388A>G polymorphisms and risk of statin-induced adverse drug reactions: A meta-analysis

**DOI:** 10.1186/s40064-016-2912-z

**Published:** 2016-08-19

**Authors:** Jiajia Jiang, Qing Tang, Jing Feng, Rong Dai, Yang Wang, Yuan Yang, Xiaojun Tang, Changkai Deng, Huan Zeng, Yong Zhao, Fan Zhang

**Affiliations:** 1 School of Public Health and Management, Research Center for Medicine and Social Development, Innovation Center for Social Risk Governance in Health, Chongqing Medical University, Chongqing, 400016 China; 2 Department of Cardiovascular Medicine, the First Affiliated Hospital of Chongqing Medical University, Chongqing, 400016 China; 3 Chengdu Women’s and Children’s Central Hospital, Chengdu, 610000 Sichuan China; 4 Department of Epidemiology, School of Public Health and Management, Chongqing Medical University, No. 1 Medical College Road, Yuzhong District, Chongqing, 400016 China

**Keywords:** SLCO1B1, Polymorphism, Statin, Adverse drug reaction, Meta-analysis

## Abstract

An increasing number of studies have investigated the association between SLCO1B1 −521T>C and −388A>G polymorphisms and the risk of statin-induced adverse drug reactions (ADRs), but the results have been inconsistent. This meta-analysis was performed to gain more insight into the relationship. PubMed, Embase, Cochrane Library and Web of Science were searched for relevant articles published before March 5th, 2015. The quality of included studies was evaluated by the Newcastle-Ottawa Quality scale. Pooled effect estimates (odds ratios [ORs] or hazard ratios [HRs) and corresponding 95 % confidence intervals (CIs) were calculated to assess the association in overall and subgroup analyses for various genetic models. Begg’s rank correlation test and Egger’s linear regression test were used to examine the publication bias. A total of nine cohort and four case–control studies involving 11, 246 statin users, of whom 2, 355 developing ADRs were included in the analysis. Combined analysis revealed a significant association between the SLCO1B1−521T>C polymorphism and increased risk for ADRs caused by various statins, but the synthesis heterogeneity was generally large (dominant model: pooled effect estimate = 1.85, 95 % CI 1.20–2.85, *P* = 0.005; *I*^2^ = 80.70 %, Pheterogeneity < 0.001). Subgroup analysis by statin type showed that the ADRs risk was significantly elevated among simvastatin users (dominant model: pooled effect estimate = 3.43, 95 % CI 1.80–6.52, *P* = 0.001; *I*^2^ = 59.60 %, Pheterogeneity = 0.060), but not among atorvastatin users. No significant relationship was found between the −388A>G polymorphism and ADRs caused by various statins (dominant model: pooled effect estimate = 0.94, 95 % CI 0.79–1.13, *P* = 0.526; *I*^2^ = 40.10 %, Pheterogeneity = 0.196). The meta-analysis suggests that SLCO1B1 −521T>C polymorphism may be a risk factor for statin-induced ADRs, especially in simvastatin therapy. Conversely, there may be no significant association for −388A>G polymorphism.

## Background

Statins or the 3-hydroxy-3-methylglutaryl coenzyme A (HMG-CoA) reductase inhibitors have become the most important pharmaceutical intervention for the primary and secondary prevention of cardiovascular diseases by lowering blood concentrations of low density lipoprotein cholesterol (LDL-C) (Baigent et al. 2005; Stewart 2013). In general, statins are safe and well tolerated, but there are still 25–50 % of patients with coronary artery disease noncompliant after 1 year’s medication mainly because of adverse drug reactions (ADRs) (Ho et al. 2008). The musculoskeletal problem is the most common intolerance, symptoms of which range from mild clinical myalgias, “incipient” myopathy, “definite” myopathy to fatal rhabdomyolysis (Feng et al. 2012; Ghatak et al. 2010; Link et al. 2008; Thompson et al. 2006). Though the rate of statin-induced myopathy is low, the absolute number of patients with statin-related muscle symptoms may be substantial due to the high prevalence of cardiovascular diseases and the wide use of statin drugs (Wilke et al. 2005a, b). In addition, many patients discontinue statin therapy for some mild myopathic symptoms, which increases their potential risk of cardiovascular events to some extent (Wilke et al. 2012). Therefore, understanding the mechanism of statin-induced ADRs has great significance to reduce statin toxicity and optimize patients’ adherence.

Previous studies showed that a series of clinical factors may contribute to the risk of developing muscle toxicity during statin therapy, including older age (though existing controversy) (Schech et al. 2007; Graham et al. 2004), low body mass index, female gender (de Keyser et al. 2014), higher dose (de Lemos et al. 2004; McClure et al. 2007), metabolic comorbidities (i.e., hypothyroidism), intense physical exercise (Meador and Huey 2010), interactions with other drugs such as fibrates, ciclosporin, protease inhibitors, macrolide antibiotics, and amio darone (Wilke et al. 2012; Graham et al. 2004; Niemi et al. 2011). In addition, genetic factors also play a vital role. It was reported that variability in statin metabolizing enzyme genes (CYP3A4/5, CYP2C8/9, CYP2D6, UGT1), membrane transporter genes (SLCO1B1, ABCB1, ABCG2) and pharmacodynamic related genes (CPT2, COQ2, RYR2) could influence the development and severity of statin-associated muscle toxicity (Feng et al. 2012; Ishikawa et al. 2004; Wilke et al. 2005a, b; Frudakis et al. 2007; Fiegenbaum et al. 2005; Vladutiu et al. 2006; Vladutiu 2008; Oh et al. 2007; Marciante et al. 2011). Among them, the SLCO1B1 gene is widely investigated. The SLCO1B1 gene locates on the chromosome 12 (Chr 12p12.2) occupying 109 kb, which encodes the organic anion transporting polypeptide 1B1 (OATP1B1). This is an influx transporter expressed on the sinusoidal membrane of human hepatocytes and facilitates the liver uptake of most statins (atorvastatin, rosuvastatin, pravastatin, simvastatin, and lovastatin) (Pasanen et al. 2007; König et al. 2006; Pasanen et al. 2006; Chen et al. 2008). Only a few are known to have functional effects in spite of many single-nucleotide polymorphisms (SNPs) have been identified in SLCO1B1. Previous studies mostly focused on the variants −521T>C (rs4149056) and −388A>G (rs2306283). It is reported that patients with the −521C minor allele had reduced hepatic uptake and increased blood concentration of various statins, which might increase the ADRs risk (Pasanen et al. 2006, 2007; Kameyama et al. 2005; Niemi et al. 2004). The role of −388A>G in the transporting activity of OATP1B1 varies in different studies (Niemi et al. 2011). In recent years, an increasing number of studies began to explore the role of SLCO1B1 polymorphisms in statin-induced ADRs. However, the results remain inconsistent and with limited power (Link et al. 2008; de Keyser et al. 2014; Marciante et al. 2011; Voora et al. 2009; Danik et al. 2013; Donnelly et al. 2011; Carr et al. 2013; Santos et al. 2012; Brunham et al. 2012).

A meta-analysis of seven studies investigating association of statin myopathy with the SLCO1B1−521T>C was published in 2013 (Carr et al. 2013). However, there were three otherwise eligible articles (de Keyser et al. 2014; Danik et al. 2013; Santos et al. 2012) not included in the analysis, which would influence the validity of the pooled estimates. Also, the publication did not present the exact literature selection criteria or the extracted data of its included original studies, thus it was difficult to check some pivotal information. We performed this meta-analysis to provide a more comprehensive estimation of the association between the two SNPs, SLCO1B1 −521T>C and −388A>G polymorphisms and statin-induced various ADRs not only myopathy, under various genetic models. The finding of a significant correlation may become useful in prestatin treatment screening in order to predict the chance of the development of adverse effects.

## Methods

### Search strategy

Four databases were electronically searched to retrieve studies on association between statin-induced ADRs and polymorphisms of the SLCO1B1 gene until 5 March 2015, including PubMed, ISI web of knowledge, Embase, and the Cochrane Library. Searching terms were: “HMG-CoA reductase inhibitor” or “statin” or “simvastatin” or “lovastatin” or “fluvastatin” or “atorvastatin” or “pravastatin” or “rosuvastatin” or “cerivastatin” or “mevastatin”, combined with “SLCO1B1”. In addition, references of the retrieved publications were checked for relevant studies.

### Inclusion and exclusion criteria

Titles and abstracts of all retrieved publications were screened. Then the full-text screening was conducted by two researchers (Jiang and Tang) independently. Any discordance was subsequently resolved through discussion or a third party (Zhang). Studies were considered eligible if they met all of the following criteria: (1) It investigated the association between −521T>C or −388A>G polymorphisms in the SLCO1B1 gene and the risk of statin-induced ADRs; (2) It provided effect estimates (OR, RR, or HR) and their corresponding 95 % confidence intervals (95 % CIs) or allele or genotype frequencies for calculating the effect estimates; (3) The publication language was English. Studies consistent with any of the following conditions were excluded: (1) Reviews, case reports, comments, letters, news, editorials; (2) In vitro or animal trials; (3) Studies lacking information necessary or usable data for the analysis; (4) For overlapping and republished studies, only the most recent or the largest population was included.

### Data extraction

Data were extracted independently by two reviewers (Jiang and Tang). Disagreements were solved by discussion, and a third party (Zhang) was involved when necessary. The collected information included: first author, year of publication, country where study was conducted, ethnicity, study design, definition of ADRs, characteristics of participants, statin type, dose and regimen, sample size, polymorphism region, allele or genotype frequencies in patients with or without ADRs during the treatment of statins, crude and adjusted effect estimates (ORs, RRs, HRs) and their corresponding 95 % CIs as reported, any multivariate analyses adjustment factors, genotyping method, and information about Hardy–Weinberg Equilibrium (HWE).

### Quality assessment of included studies

Newcastle-Ottawa Quality scale (NOS) (Crowther et al. 2010; Cota et al. 2013) was developed for quality assessment. A “star system” (range 0–9 stars) was used for the present analysis to evaluate each included study on the following: selection of the study groups, between-group comparability, and the ascertainment of either the exposure for case–control studies or the outcome for cohort studies. A study can be awarded a maximum of one star for each numbered item within the Selection and Exposure or Outcome categories. A maximum of two stars can be assigned for Comparability. With respect to the follow-up period sufficient for outcomes to occur, the minimum follow-up of the exposed group was set at 1 year, given that the risk of an adverse drug reaction is greater during the first year of therapy, and decreases afterwards (de Keyser et al. 2014).

### Statistical analysis

The effect estimates that were extracted, if available, or de novo calculated from available data, were crude and adjusted ORs and HRs. We pooled OR estimates and HR estimates for each study together. If both the crude and the adjusted effect estimates were available for the same outcome, we incorporated the adjusted one into the analysis. Pooled effect estimates were calculated for all of the following genetic models: the allele contrast, the homozygote comparison, the heterozygote comparison, the dominant model, the recessive model, and the additive model respectively. Heterogeneity among included studies was assessed by Chi square-based *Q* test and *I*^2^ test (Higgins and Thompson 2002). If the data showed no heterogeneity (*P* > 0.10, *I*^2^ < 50 %), the Mantel–Haenszel fixed effect model was used (Mantel and Haenszel 1959), otherwise the DerSimonian-Laird random effect model was used (DerSimonian and Laird 1986). In addition, stratification analysis was conducted by the type of statin. If the original research did not provide information about HWE, we calculated it using an online HWE calculation tool (Rodriguez et al. 2009). Sensitivity analysis was carried out to investigate the influence of a single study on the overall risk by omitting one study each time and was conducted based on leave-one-out sensitivity procedure. Publication bias was assessed by the Begg’s rank correlation test and Egger’s linear regression test if the number of included studies in the meta-analysis was more than two (Begg and Mazumdar 1994; Egger et al. 1997). Data were analyzed using the STATA 12.0 (Stata Statistical Software, College Station, TX, USA, www.stata.com) software. A *P* value of 0.05 for any test or model was considered to be statistically significant.

## Results

### Literature search

**Fig. 1:**
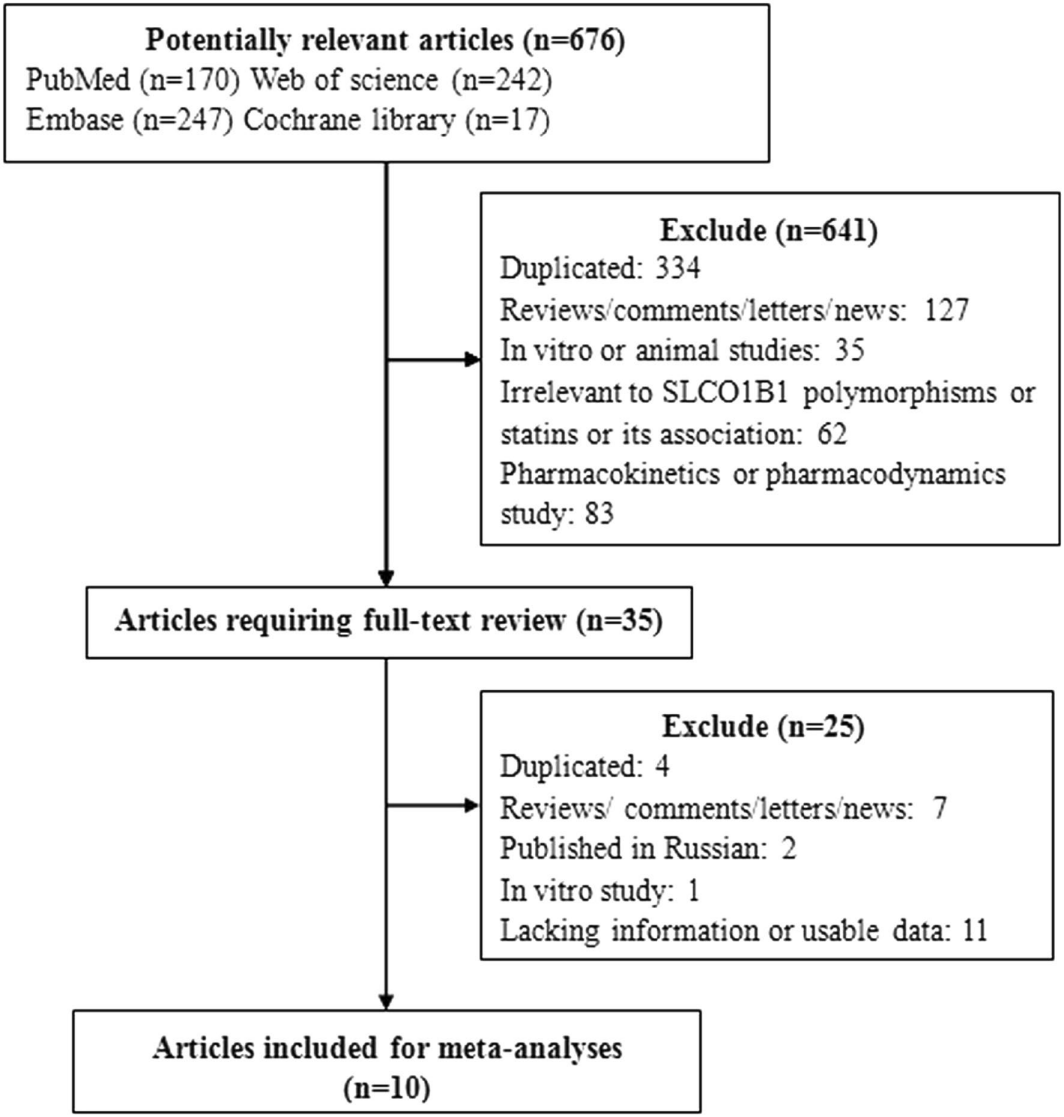
Flow chart of study selection. *n* Number of studies

A total of 676 records were yielded by the search strategy from four electronic databases. Among these, 35 publications met the inclusion criteria after title and abstract screening. Based on full-text inspection, we excluded another 25 publications because 11 of them lacked information necessary or usable data for the analysis (Dlouha et al. 2013; Dmitry et al. 2013; Mafalda et al. 2013; Pranculis and Kucinskas 2013; Hopewell et al. 2012; Khan et al. 2011; Johansen et al. 2010; Tamraz et al. 2013; Toms et al. 2010; Kamatani and Mushiroda 2011; Kroemer 2010); seven articles were reviews (three articles) (Patel et al. 2014; de Keyser et al. 2012a, b; Dendramis 2011), comments (two articles) (Wright et al. 2011; Nakamura 2008), news (one article) (Dolgin 2013) or letters (one article) (Puccetti et al. 2010); four articles reported overlapping data for the same study (de Keyser et al. 2012a, b; Toms et al. 2009; Danik et al. 2012; Voora et al. 2008); two studies were published in Russian (Sychev et al. 2013; Petrov et al. 2013) and one study was an in vitro study (Furihata et al. 2009). Among the 10 publications included, one (de Keyser et al. 2014) described four independent studies. Of these, two investigated simvastatin-related ADRs and the remaining two investigated atorvastatin-related ADRs. Thus, 13 studies from 10 publications (Link et al. 2008; de Keyser et al. 2014; Marciante et al. 2011; Voora et al. 2009; Danik et al. 2013; Donnelly et al. 2011; Carr et al. 2013; Santos et al. 2012; Brunham et al. 2012; Linde et al. 2010) were included in the meta-analysis. Details of the study selection process are presented in Fig. 1.

### Study characteristics

Characteristics of the eligible studies are summarized in Table 1. All studies were published between 2008 and 2014, with a total number of 11, 246 subjects, of whom 2, 355 developing ADRs. Five studies were conducted in the Netherlands, three in the UK, two in the US, one in Brazil, one in the US and Canada and one in 26 countries. There were 11 studies entirely or predominantly involving Caucasian participants and two not reporting the ethnicity. Nine were cohort studies and four were case–control studies. Definitions of statin-induced ADRs, doses and treatment periods were heterogeneous among these studies. There were four hospital-based and nine population-based studies. Among the included 13 studies, three focused on simvastatin, three on atorvastatin, one on rosuvastatin, one on cerivastatin, two on mixed statins and three on both mixed statins and specific statin. Seven studies provided genotype frequencies which helped us to calculate the crude ORs under various genetic models. Nine studies reported adjusted effect estimates in some genetic models.

**Table 1:** Characteristics of included studies

Study (first author, year)	Country	Ethnicity	Study design	Definition of adverse drug reactions	Population disease	Source of participants
Voora et al. (2009)	USA	Caucasian 86 %; African American 5 %; other 9 %	Cohort	A composite adverse event (CAE) of premature discontinuation for any side effect or myalgia/muscle cramps (irrespective of CK values) or CK > 3×ULN (irrespective of symptoms)	Hypercholesterolemia	Hospital
Danik et al. (2013)	26 countries	Caucasian 100 %	Cohort	Clinical myalgia or the broader categories of muscle weakness, stiffness, or pain and clinically severe myopathy (frank myopathy and rhabdomyolysis)	No disease	Population
Donnelly et al. (2011)	UK (Scotland)	NA	Cohort	Intolerance defined as composite of abnormal CK measure 1–3 × ULN, with no abnormal recorded before statin commencement; or an abnormal ALT measure, with no abnormal before statin commencement (≥50 % increase in ALT from baseline also considered abnormal)and a relevant change in prescribing (switching statin to equivalent or lower dose, dose reduction of same statin, or discontinuation of statin prescribing)	Type 2 diabetes	Population
Link et al. (2008)	UK	Caucasian 100 %	Case–control	“Definite” myopathy: muscle symptoms, with CK > 10 × ULN; “incipient” myopathy: CK > 3×ULN and 5 × baseline level, plus AAT > 1.7 × the baseline value without an elevated AAT level alone at any other visit irrespective of muscle symptoms	Myocardial infarction	Population
de Keyser et al. (2014)	Netherlands	Caucasian 100 %	Cohort	The occurrence of either a dose decrease or a switch to another cholesterol-lowering drug or a too strong reduction in cholesterol level	NA	Population
de Keyser et al. (2014)	Netherlands	Caucasian 100 %	Cohort	The occurrence of either a dose decrease or a switch to another cholesterol-lowering drug or a too strong reduction in cholesterol level	NA	Population
de Keyser et al. (2014)	Netherlands	Caucasian 98 %	Cohort	The occurrence of either a dose decrease or a switch to another cholesterol-lowering drug or a too strong reduction in cholesterol level	Hypercholesterolemia and/or hypertension	Population
de Keyser et al. (2014)	Netherlands	Caucasian 98 %	Cohort	The occurrence of either a dose decrease or a switch to another cholesterol-lowering drug or a too strong reduction in cholesterol level	Hypercholesterolemia and/or hypertension	Population
Carr et al. (2013)	UK	Caucasians 100 %	Case–control	Myopathy: CK > 4×ULN; severe phenotype:denoted by CPK > 10 × ULN or rhabdomyolysis	Type2 diabetes, Alcohol dependence, Asthma, hypertention et al.	Population
Santos et al. (2012)	Brazil	Caucasian 87 %; Mulatto 10 %; African 3 %	Cohort	Myalgia defined as muscle pain irrespective of CK values or CK > 3×ULN irrespective of symptoms	Familial hypercholesterolemia	Hospital
Linde et al. (2010)	USA	NA	Cohort	Myalgias defined as muscular pain or weakness as reported by the patients, who graded their symptoms as mild, moderate or severe	Endocrine disorders	Hospital
Marciante et al. (2011)	Case:US, Canada; control:US	Caucasian for case, control 1 (CHS), control 2 (HVH): 90.8, 84.5, 89.4 %	Case–control	Definite rhabdomyolysis defined as muscle pain or weakness associated with CK > 10 × ULN	Dyslipidemia	Population
Brunham et al. (2012)	Netherlands	Caucasian100 %	Case–control	Plasma CK > 10 × ULN.	Dyslipidemia	Hospital

Study (first author, year)	Statin type	Dose/regimen [mean (SD)] (mg/d)	SNP	Case/(control or cohort size)	Case			Control		
					TT	TC	CC	TT	TC	CC
Voora et al. (2009)	Mixed	Multiple doses	rs4149056	97/351	62	31	4	263	84	4
	Simvastatin	20.0 for 8 weeks + 80.0 for 8 weeks		34/124	17	17^b^		91	33^b^	
	Atorvastatin	10.0 for 8 weeks + 80.0 for 8 weeks		31/115	21	10^b^		88	27^b^	
	Pravastatin	10.0 for 8 weeks + 40.0 for 8 weeks		31/111	23	8^b^		83	28^b^	
Danik et al. (2013)	Rosuvastatin	20.0	rs4149056	471/4404^a^	NA	NA	NA	NA	NA	NA
Donnelly et al. (2011)	Mixed (simvastatin major)	Multiple doses	rs4149056	816/1275	565	227	24	905	348	22
			rs2306283	816/1275	316	399	101	471	606	198
Link et al. (2008)	Simvastatin	80.0	rs4149056	85/90	29	35	21	70	17	3
de Keyser et al. (2014)	Simvastatin	Starting dose:20.0(11.4)	rs4149056	319/1462^a^	NA	NA	NA	NA	NA	NA
de Keyser et al. (2014)	Atorvastatin	Starting dose:17.8(13.2)	rs4149056	110/367^a^	NA	NA	NA	NA	NA	NA
de Keyser et al. (2014)	Simvastatin	Starting dose:41.7(18.9)	rs4149056	88/393^a^	NA	NA	NA	NA	NA	NA
de Keyser et al. (2014)	Atorvastatin	Starting dose:45.0(34.8)	rs4149056	42/244^a^	NA	NA	NA	NA	NA	NA
Carr et al. (2013)	Mixed	Case:33.2 (15.7), contro:30.6(15.7)	rs4149056	76/372	40	30	6	260	101	11
	Simvastatin	NA		59/222	29	25	5	147	71	4
	Atorvastatin	NA		11/110	7	4	0	86	22	2
Santos et al. (2012)	Atorvastatin	Case:62.9(20.5) control:61.0(22.7)	rs4149056	14/129	12	2^b^		94	35^b^	
			rs2306283	14/129	10	4^b^		70	59^b^	
Linde et al. (2010)	Mixed	Multiple doses	rs4149056	27/19	14	12	1	15	4	0
Marciante et al. (2011)	Cerivastatin	Case:0.6(0.2)	rs4149056	65/716	NA	NA	NA	NA	NA	NA
			rs2306283	185/732	NA	NA	NA	NA	NA	NA
Brunham et al. (2012)	Mixed	Case:31.0 (23.0) control:36.0 (25.0)	rs4149056	25/83	15	8	2	57	20	6
	Simvastatin	NA		12/39	5	6	1	27	10	2
	Atorvastatin	NA		10/34	7	2	1	24	7	3

Study (first author, year)	Effect estimate (95 % CI)						
	CC vs. TT	TC vs. TT	TC/CC vs. TT	CC vs. TT/TC	Additive	C vs. T	Adjusting factors
Voora et al. (2009)	4.24 (1.03–17.43)^c^	1.57 (0.95–2.57)^c^	1.70 (1.04–2.80)^e^	3.73 (0.92–15.20)^c^	1.70 (1.11–2.61)^c^	1.67 (1.10–2.52)^c^	Race, gender
	NA	NA	2.76 (1.26–6.02)^c^	NA	NA	NA	–
	NA	NA	1.55 (0.65–3.70)^c^	NA	NA	NA	–
	NA	NA	1.03 (0.41–2.57)^c^	NA	NA	NA	–
Danik et al. (2013)	1.13 (0.65–1.97)^d^	0.90 (0.72–1.12)^d^	NA	NA	0.95 (0.79–1.15)^d^	NA	–
Donnelly et al. (2011)	1.75 (0.97–3.15)^c^	1.04 (0.86–1.27)^c^	1.09 (0.90–1.32)^c^	2.05 (1.02,4.09)^e^	1.12 (0.94–1.33)^c^	1.12 (0.94–1.32)^c^	Statin adherence, study duration, maximum dose, prescription for other lipid–regulating drugs, cyp3a4 inhibiting drugs, age, and rs2306283
	0.76 (0.58–1.01)^c^	0.98 (0.81–1.19)^c^	0.93 (0.77–1.11)^c^	0.71 (0.52,0.96)^e^	0.90 (0.79–1.02)^c^	0.90 (0.79–1.02)^c^	Statin adherence, study duration, maximum dose, prescription for other lipid–regulating drugs, cyp3a4 inhibiting drugs, age, and rs4149056
Link et al. (2008)	16.90 (4.70–61.10)	4.97 (2.41–10.24)^c^	6.76 (3.46–13.20)^c^	9.52 (2.72–33.28)^c^	4.50 (2.60–7.70)	5.65 (3.32–9.62)^c^	–
de Keyser et al. (2014)	1.74 (1.05–2.88)^d,e^	0.77 (0.58–1.02)^d,e^	NA	NA	NA	NA	Age, sex, and starting dose
de Keyser et al. (2014)	1.49 (0.54–4.10)^d,e^	1.17 (0.77–1.79)^d,e^	NA	NA	NA	NA	Age, sex, and starting dose
de Keyser et al. (2014)	1.38 (0.34–5.71)^d,e^	0.74 (0.45–1.24)^d,e^	NA	NA	NA	NA	Age, sex, and starting dose
de Keyser et al. (2014)	NA	NA	0.97 (0.68–1.40)^d,e^	NA	NA	NA	Age, sex, and starting dose
Carr et al. (2013)	4.32 (1.82–10.43)^e^	1.93 (1.14–3.27)^c^	2.09 (1.27–3.45)^c^	2.81 (1.01–7.86)^c^	2.08 (1.35–3.23)^e^	1.93 (1.29–2.89)^c^	Statin type,previous history of type 2 diabetes, asthma,hypertension
	6.33 (1.60–25.02)^c^	1.78 (0.97–3.27)^c^	2.03 (1.13–3.63)^c^	5.05 (1.31–19.43)^c^	2.13 (1.29–3.54)^e^	1.95 (1.23–3.10)^c^	
	2.31 (0.10–52.59)^c^	2.23 (0.60–8.32)^c^	2.05 (0.55–7.58)^c^	1.89 (0.09–41.74)^c^	1.91 (0.56–6.54)^e^	1.66 (0.52–5.28)^c^	
Santos et al. (2012)	NA	NA	2.24 (0.47–10.72)^e^	NA	NA	NA	NA
	NA	NA	2.08 (0.62–7.00)^e^	NA	NA	NA	NA
Linde et al. (2010)	3.21 (0.12–85.20)^c^	3.21 (0.84–12.35)^c^	3.48 (0.92–13.25)^c^	2.21 (0.09–57.14)^c^	3.44 (0.95–12.53)^c^	2.98 (0.90–9.89)^c^	–
Marciante et al. (2011)	NA	NA	NA	NA	2.45 (1.61–3.75)^e^	NA	Age at statin use, sex, and race
	NA	NA	NA	NA	0.96 (0.75,1.24)^e^	NA	
Brunham et al. (2012)	1.27 (0.23–6.92)^c^	1.52 (0.56–4.12)^c^	1.50 (0.58–3.69)	1.12 (0.21–5.91)^c^	1.26 (0.63–2.51)^c^	1.32 (0.62–2.81)^c^	–
	2.70 (0.20–35.75)^c^	3.24 (0.81–13.02)^c^	4.50 (0.73–27.59)	1.68 (0.14–20.35)^c^	2.19 (0.79–6.12)^c^	2.29 (0.82–6.38)^c^	–
	1.14 (0.10–12.79)^c^	0.98 (0.17–5.83)^c^	1.06 (0.22–4.80)	1.15 (0.11,12.43)^c^	1.04 (0.36–3.07)^c^	1.06 (0.30–3.70)^c^	–

Study (first author, year)	Statin type	Genotyping methods	Deviation from HWE
Voora et al. (2009)	Mixed	NA	No
	Simvastatin		NA
	Atorvastatin		NA
	Pravastatin		NA
Danik et al. (2013)	Rosuvastatin	NA	NA
Donnelly et al. (2011)	Mixed (simvastatin major)	TAQMAN assays	No
			No
Link et al. (2008)	Simvastatin	PCR-fluorescence	No
de Keyser et al. (2014)	Simvastatin	Microarray genotyping procedures	NA
de Keyser et al. (2014)	Atorvastatin	Microarray genotyping procedures	NA
de Keyser et al. (2014)	Simvastatin	TaqMan allelic discrimination	NA
de Keyser et al. (2014)	Atorvastatin	TaqMan allelic discrimination	NA
Carr et al. (2013)	Mixed	TaqMan real-time PCR SNP genotyping assays	No
	Simvastatin		No
	Atorvastatin		No
Santos et al. (2012)	Atorvastatin	PCR-HRM	No
			No
Linde et al. (2010)	Mixed	PCR and DNA sequencing	No
Marciante et al. (2011)	Cerivastatin	Illumina Goldengate custom panel; Taqman 5′ nuclease discrimination assay; pyrosequencing on the PyroMark Q96MD platform.	NA
			NA
Brunham et al. (2012)	Mixed	Illumina Goldengate genotyping assay	Yes
	Simvastatin		No
	Atorvastatin		No

*CI* confidence interval, *NA* not available, *HWE* Hardy–Weinberg equilibrium, *PCR* polymerase chain reaction

^a^Case/size of cohort; ^b^ The frequencey of TC/CC; ^c^ The estimates were calculated using the original data; ^d^ Hazard ratio (HR); ^e^ The adjusted effect estimates

### Quality of included studies

Rating of the quality of the included studies according to the NOS is presented in Table 2. The quality scores ranged from 5 to 9. The case definition was adequate in all of the included case–control studies, but the non-response rate between the case and control groups was equal in only one study (Brunham et al. 2012). For all cohort studies, 4 stars were assigned to Selection category. The follow-up time for exposed participants was not reported in the study by Linde et al. (2010) and not long enough (<1 year) for the outcome to occur in the study by Voora et al. (2008). Nearly all included cohort studies did not mention the loss to follow-up.

**Table 2:** Methodological quality assessment of included studies based on the Newcastle–Ottawa Scale

Author (years)	Study design	Selection				Comparability	Exposure/outcome			Total
		1	2	3	4	5	6	7	8	
Link et al. (2008)	Case–control	*	*	*	*	**	*	*		********
Carr et al. (2013)	Case–control	*	*	*	*	*	*	*		*******
Marciante et al. (2011)										
	Case–control	*			*	*	*	*		*****
Brunham et al. (2012)	Case–control	*	*	*	*	**	*	*	*	*********
Voora et al. (2009)	Cohort	*	*	*	*	*	*		*	*******
Danik et al. (2013)	Cohort	*	*	*	*	*	*	*		*******
Donnelly et al. (2011)	Cohort	*	*	*	*	*	*	*		*******
de Keyser et al. (2014)	Cohort	*	*	*	*	**	*	*		********
de Keyser et al. (2014)	Cohort	*	*	*	*	**	*	*		********
de Keyser et al. (2014)	Cohort	*	*	*	*	**	*	*		********
de Keyser et al. (2014)	Cohort	*	*	*	*	**	*	*		********
Santos et al. (2012)	Cohort	*	*	*	*	*	*	*	*	********
Linde et al. (2010)	Cohort	*	*	*	*	*				*****

### Meta-analysis results

**Fig. 2:**
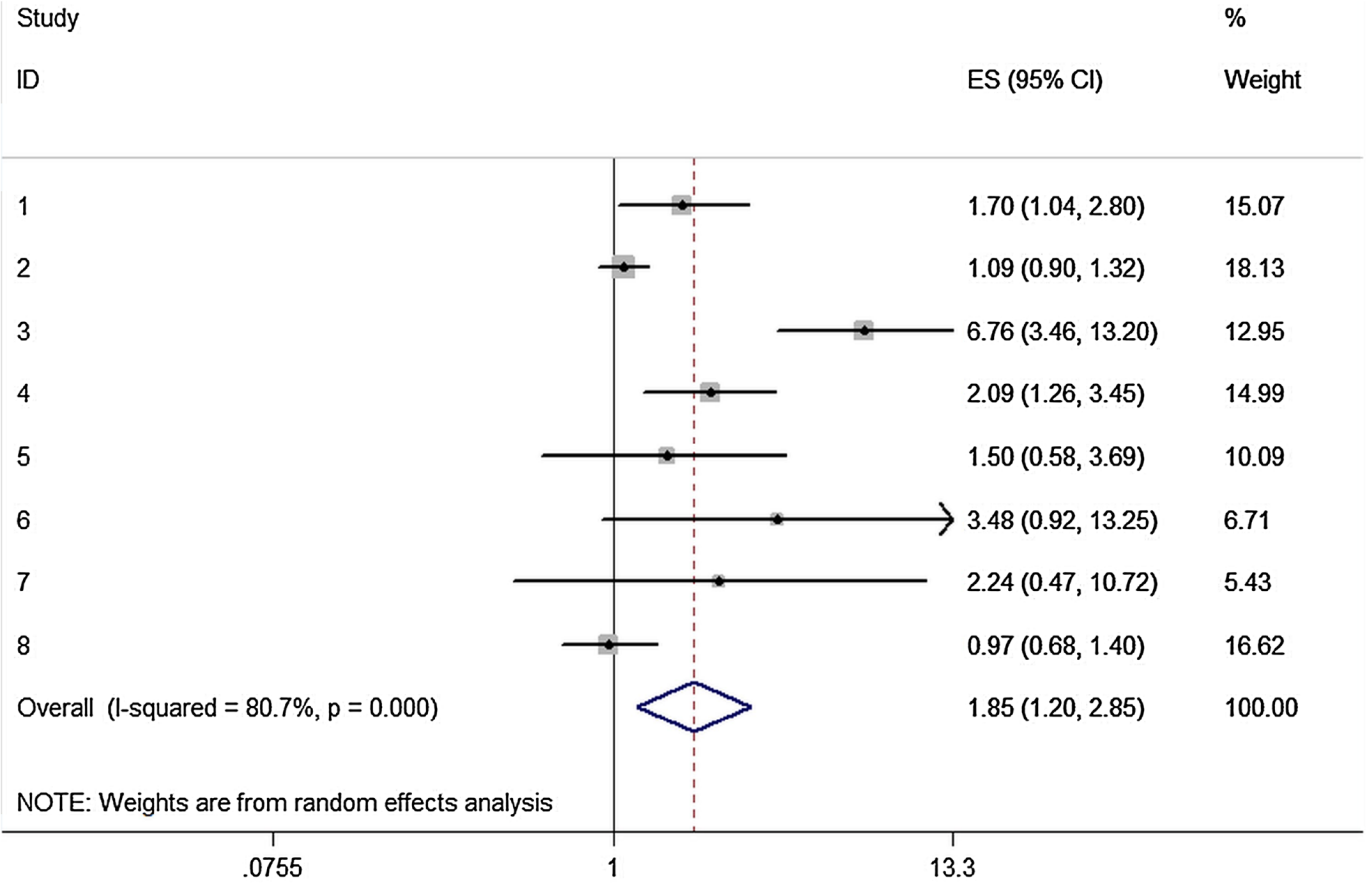
Association between *SLCO1B1* −521T>C polymorphism and risk of adverse drug reactions caused by any statin. Dominant genetic model (TC/CC vs. TT). The ES is odds ratio (OR) or hazard ratio (HR); the size of the square is proportional to the weight of each study; *horizontal lines* represent the 95 % CI

All of the 13 included studies explored the association between −521T>C polymorphism of the SLCO1B1 gene and statin-induced ADRs. The results of meta-analysis are presented in Table 3. A significant increased risk of ADRs induced by different types of statins was found for the C vs. T: pooled effect estimate = 1.99, 95 % CI 1.20–3.29, *P* = 0.007); the homozygote comparison (CC vs. TT: pooled effect estimate = 2.21, 95 % CI: 1.41–3.47, *P* = 0.001); the dominant model (TC/CC vs. TT: pooled effect estimate = 1.85, 95 % CI 1.20–2.85, *P* = 0.005); the recessive model (CC vs. TT/TC: pooled effect estimate = 2.76, 95 % CI 1.73–4.39, *P* < 0.001) and the additive model (per C allele: pooled effect estimate = 1.76, 95 % CI 1.25–2.50, *P* = 0.001), but not for the heterozygote comparison (TC vs. TT: pooled effect estimate = 1.26, 95 % CI 0.96–1.65, *P* = 0.091). Figure 2 shows the pooled effect estimate and 95 % CI for any statin induced ADRs in the dominant model.

**Table 3:** Association between −521T>C and −388A>G polymorphisms in *SLCO1B1* gene and statin-induced adverse reactions risk

Polymorphism	Statin type	Genetic model	N	Effect estimate (95 % CI)	Z	*P* value	*I* ^2^ %	P_het_	Effect model	Begg’s test		Egger’s test	
										z	P	t	P
−521T>C	Statins	C vs T	6	1.99 (1.20–3.29)	2.69	0.007	86.90 %	<0.001	R	0.38	0.707	1.91	0.129
		CC vs TT	10	2.21 (1.41–3.47)	3.45	0.001	55.20 %	0.017	R	0.54	0.592	1.32	0.223
		TC vs TT	10	1.26 (0.96–1.65)	1.69	0.091	75.80 %	<0.001	R	1.97	0.049	2.36	0.046
		TC/CC vs TT	8	1.85 (1.20–2.85)	2.80	0.005	80.70 %	<0.001	R	1.11	0.266	2.15	0.075
		CC vs TT/TC	6	2.76 (1.73–4.39)	4.27	<0.001	13.60 %	0.327	F	0.00	1.000	0.31	0.769
		Additive	8	1.76 (1.25–2.50)	3.19	<0.001	86.10 %	<0.001	R	0.87	0.386	2.97	0.025
	Simvastatin	C vs T	3	3.00 (1.39–6.48)	2.80	0.005	77.90 %	0.011	R	0.00	1.000	0.01	0.993
		CC vs TT	5	3.62 (1.33–9.83)	2.52	0.012	69.10 %	0.012	R	–0.24	1.000	1.06	0.367
		TC vs TT	5	1.58 (0.78–3.19)	1.28	0.200	86.50 %	<0.001	R	1.22	0.221	2.07	0.130
		TC/CC vs TT	4	3.43 (1.80–6.52)	3.76	<0.001	59.60 %	0.060	R	0.34	0.734	0.32	0.777
		CC vs TT/TC	3	5.98 (2.53–14.13)	4.07	<0.001	0.00 %	0.453	F	1.04	0.296	–2.06	0.288
		Additive	3	2.87 (1.67–4.94)	3.81	<0.001	52.80 %	0.120	R	0.00	1.000	–0.17	0.892
	Atorvastatin	C vs T	2	1.35 (0.58–3.15)	0.69	0.491	0.00 %	0.605	F	–	–	–	–
		CC vs TT	3	1.49 (0.61–3.65)	0.87	0.383	0.00 %	0.941	F	0.00	1.000	0.23	0.858
		TC vs TT	3	1.23 (0.83–1.82)	1.03	0.303	0.00 %	0.635	F	0.00	1.000	0.52	0.697
		TC/CC vs TT	5	1.11 (0.82–1.52)	0.69	0.492	0.00 %	0.606	F	0.73	0.462	2.60	0.081
		CC vs TT/TC	2	1.38 (0.21,9.13)	0.33	0.738	0.00 %	0.803	F	–	–	–	–
		Additive	2	1.36 (0.60–3.05)	0.74	0.460	0.00 %	0.468	F	–	–	–	–
	Rosuvastatin	CC vs TT	1	1.13 (0.65–1.97)	–	–	–	–	–	–	–	–	–
		TC vs TT	1	0.90 (0.72–1.12)	–	–	–	–	–	–	–	–	–
		Additive	1	0.95 (0.79–1.15)	–	–	–	–	–	–	–	–	–
	Pravastatin	TC/CC vs TT	1	1.03 (0.41–2.57)	–	–	–	–	–	–	–	–	–
	Cerivastatin	Additive	1	2.45 (1.61–3.75)	–	–	–	–	–	–	–	–	–
−388A>G	Statins	TC/CC vs TT	2	0.94 (0.79–1.13)	0.63	0.526	40.10 %	0.196	F	–	–	–	–
		Additive	2	0.91 (0.81–1.02)	1.58	0.114	0.00 %	0.649	F	–	–	–	–

*N* number of included studies in the meta-analysis, *CI* confidence interval, *P*_het_, *P* value of heterogeneity, *F* fixed-effect model, *R* random-effect model

**Fig. 3:**
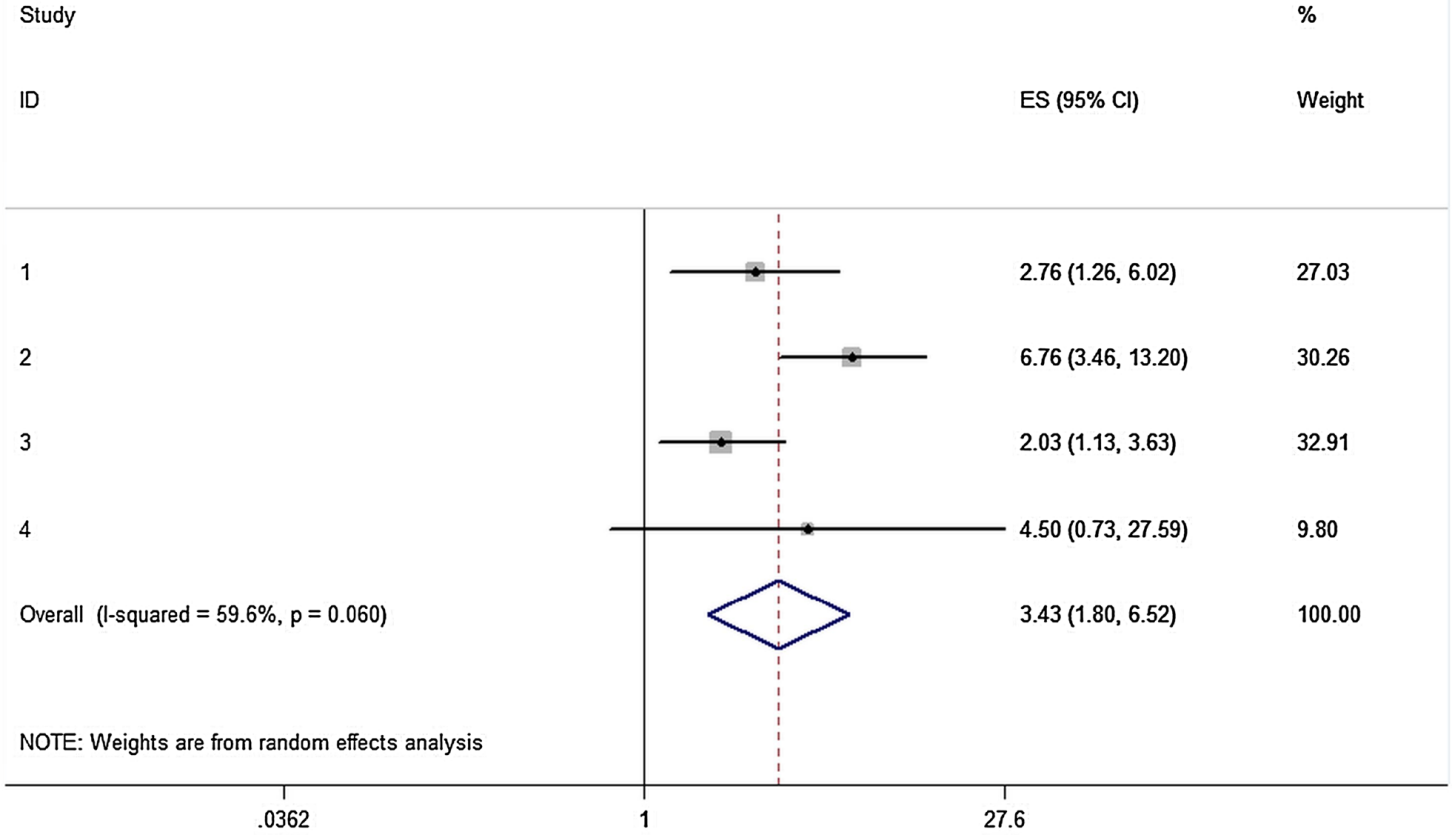
Association between *SLCO1B1* −521T>C polymorphism and risk of adverse drug reactions caused by simvastatin. Dominant genetic model (TC/CC vs. TT). The ES is odds ratio (OR); the size of the square is proportional to the weight of each study; *horizontal lines* represent the 95 % CI

**Fig. 4:**
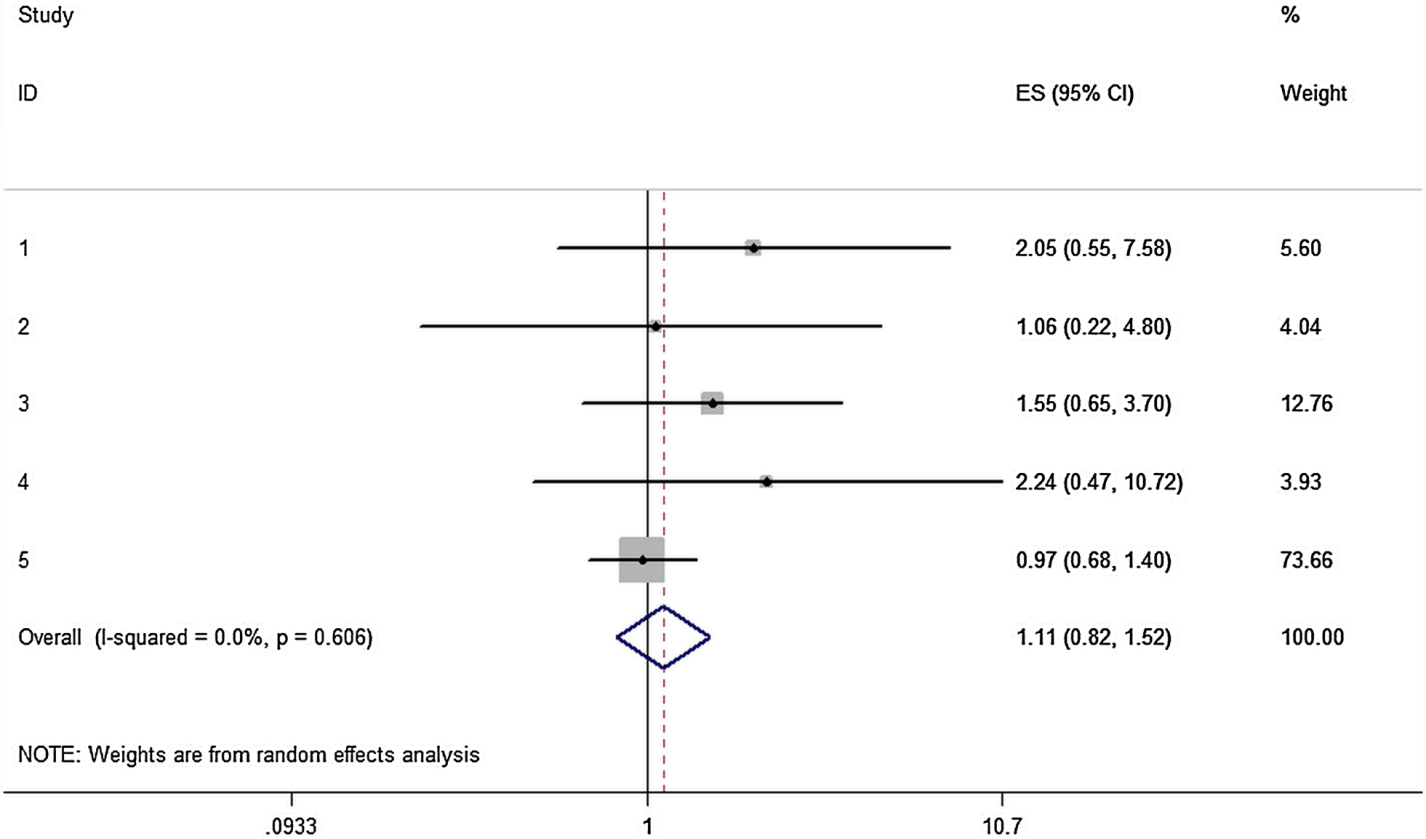
Association between *SLCO1B1* −521T>C polymorphism and risk of adverse drug reactions caused by atorvastatin. Dominant genetic model (TC/CC vs. TT). The ES is odds ratio (OR) or hazard ratio (HR); the size of the square is proportional to the weight of each study; *horizontal lines* represent the 95 % CI

In the stratified analysis by statin type (Table 3), the synthesis of studies investigating simvastatin pointed to a statistically significant increased risk for the allele contrast model (C vs. T: pooled effect estimate = 3.00, 95 % CI 1.39–6.48, *P* = 0.005), the homozygote comparison (CC vs. TT: pooled effect estimate = 3.62, 95 % CI 1.33–9.83, *P* = 0.012), the dominant model (TC/CC vs. TT: pooled effect estimate = 3.43, 95 % CI 1.80–6.52, *P* < 0.001) (Fig. 3), the recessive model (CC vs. TT/TC: pooled effect estimate = 5.98, 95 % CI 2.53–14.13, *P* < 0.001) and the additive model (per C allele: pooled effect estimate = 2.87, 95 % CI 1.67–4.94, *P* < 0.001). Whereas in contrast, the combined effect estimates for atorvastatin-induced ADRs were far from being statistically significant in any of the six genetic models. The association between the −521T>C polymorphism and atorvastatin-induced ADRs in the dominant model is shown in Fig. 4.

Three of the included studies explored the relationship between the −388A>G polymorphism of the SLCO1B1 gene and statin-induced ADRs. Due to the limited available data from the original studies, we only performed meta-analysis under the dominant model and the additive model based on two studies respectively. Both of the results yielded a decreased risk for statin-related ADRs among −388G allele carriers, although the pooled effect estimates did not reach statistical significance (AG/GG vs. AA: pooled effect estimate = 0.94, 95 % CI 0.79–1.13, *P* = 0.526; the additive model: pooled effect estimate = 0.91, 95 % CI 0.81–1.02, *P* = 0.114) (Table 3).

### Sensitivity analysis

For −521T>C polymorphism and risk of ADRs induced by any statin, the conclusion of insignificant association for the heterozygote comparison was materially altered by removing the study by de Keyser et al. (2014) with the overall effect estimates changing to 1.36 (95 % CI 1.02–1.84, *P* = 0.038) and 1.34 (95 % CI: 1.01–1.79, *P* = 0.044) respectively. In addition, the result changed and became insignificant when we excluded the study by Carr et al. (2013) in the homozygote comparison for −521T>C polymorphism and simvastatin-related ADRs (pooled effect estimate = 3.17, 95 % CI 0.96–10.53, *P* = 0.059). The results were not materially altered for other analysis except for the above situations.

### Publication bias

The Begg’s rank correlation test and Egger’s linear regression test indicated no evidence of publication bias among studies testing −521T>C and −388A>G polymorphisms of the SLCO1B1 gene and the risk of ADRs induced by various statins or specific statin (*P* > 0.05 for all of the genetic models) except in the analysis of −521T>C polymorphism and ADRs induced by any statin in the heterozygote comparison (Begg’s, *P* = 0.049; Egger’s, *P* = 0.046) and the additive model (Begg’s, *P* = 0.386; Egger’s, *P* = 0.025) (Table 3).

## Discussion

ADRs give rise to the discontinuation of statin treatment (Simons et al. 1996). Recent studies showed that genetic variants in the SLCO1B1 gene modified the risk of statin-induced myopathy, but the results were controversial. The meta-analysis aimed to retrieve all published relative articles to identify the associations between the two functional variants −521T>C and −388A>G of the SLCO1B1 gene and the risk of ADRs during statin therapy. We included articles about any ADR and the dose decrease, discontinuation or switches to other cholesterol-lowering drugs related to statin toxicity as indicators of ADRs.

The results of the meta-analysis demonstrated that the minor allele of the −521C significantly increased the risk of ADRs caused by various statins, which is consistent with that of one previous meta-analysis (Carr et al. 2013). However, the synthesis heterogeneity was generally large under the different genetic models except for the recessive model. So we could not draw a strong conclusion that the association between the rs4149056 polymorphism and the ADRs induced by statins was a class effect. Upon closer review of each selected original study, it was found that results according to different statin types were inconsistent. Thus, we conducted a stratification analysis to explore that.

After stratification analysis by the statin type, significant associations between the −521T>C polymorphism and ADRs among simvastatin users for the allele contrast, the homozygote comparison, the dominant model, the recessive model and the additive model were observed, while no significant effect was found among patients treated with atrovastatin in any of the six genetic models. The results were in line with those of one previous meta-analysis by Carr et al. (2013), in which patients who carried at least one minor allele showed a more than three-fold higher risk for simvastatin-induced side effects compared with the reference TT genotype, whereas in contrast, the relationship did not reach statistical significance within atorvastatin users. The results from the original studies indicated that the −521C minor allele was not a risk factor for rosuvastatin-induced myalgia (HR = 0.95, 95 % CI 0.79–1.15 per allele, *P* = 0.59) (Danik et al. 2013) or pravastatin-induced composite adverse events (TC/CC vs. TT: OR = 1.03, 95 % CI 0.41–2.57, *P* = 0.948) (Voora et al. 2009). On the contrary, an additional copy of the minor allele was associated with the risk of cerivastatin-associated rhabdomyolysis (OR = 2.45, 95 % CI 1.61–3.75, *P* = 3.11E−05) (Marciante et al. 2011). However, as there was only one study investigating cerivastatin, rosuvastatin and pravastatin respectively, we did not perform a meta-analysis to provide evidence for their relationship.

There remains uncertainty about the biological mechanisms underlying the statin-associated myopathy but statin concentrations in the blood was one possible reason (Stewart 2013). A non-synonymous coding SNP, rs4149056 in the SLCO1B1 gene encodes a valine-to-alanine substitution that leads to a less active form of the OATP1B1 transporter and hence increases the plasma drug concentration (Kameyama et al. 2005; Tirona et al. 2001; Nozawa et al. 2002; Iwai et al. 2004). A previous study showed that in transient expression systems of HEK293 and HeLa cells using statins as substrates, the transporting activities of those expressing SLCO1B1 −521C allele decreased significantly (Kameyama et al. 2005). Furthermore, there are differences in the effects of SLCO1B1 −521T>C polymorphism on the pharmacokenetics of various statins. In patients with homozygous minor allele genotype, the observed plasma areas under the curve of active simvastatin acid, pitavastatin, atorvastatin, pravastatin, and rosuvastatin have been 221, 162–191, 144, 57–130, and 62–117 % higher respectively than that in patients with the wild-type genotype (Wilke et al. 2012). This may partially account for the disparate roles of rs4149056 variant in the development of ADRs during different types of statin therapy. Several case–control (Link et al. 2008; de Keyser et al. 2014; Carr et al. 2013) and cohort studies (de Keyser et al. 2014) as well as our meta-analysis confirmed the positive correlation between −521C minor allele and simvastatin-induced adverse effects. Link et al. (2008) observed that the OR for myopathy of 4.5 (95 % CI 2.6–7.7, *P* = 2 × 10^−9^) per copy of the C allele in patients taking 80 mg of simvastatin daily in the SEARCH study was approximately 2 times of that in those taking 40 mg daily within the Heart Protection Study (OR = 2.6, 95 % CI 1.3–5.0, *P* = 0.004). Besides, Carr et al. (2013) discovered in the stratification analysis for dose (a cut-off point of 40 mg), the significant increased risk associated with rs4149056 variant was only found in patients receiving ≥ 40 mg/day simvastatin. These provided evidence for dose-genotype interaction in simvastatin treatment. Consequently, the Clinical Pharmacoenomics Implementation Consortium (CPIP) Guideline for SLCO1B1 and Simvastatin-Induced Myopathy (2012) (Wilke et al. 2012) recommends that prescribing physicians should be alerted to the FDA advice on avoiding high-dose simvastatin. In our analyses, all of extracted or de novo effect estimates for atrovastatin-related ADRs showed a consistent and no significant association. While in one of the included studies by de Keyser et al. (2014), an association between the −521T>C polymorphism and dose decrease or switching to another cholesterol-lowering drug in the highest-dose category (> 20 mg) was found (the adjusted HR = 3.26, 95 % CI: 1.47–7.25, *P* = 0.004), however, we extracted the HR for whole population, which showed no significance, other than the outcome of stratification analysis. Therefore, we could anticipate that a significant higher risk would appear if patients are prescribed to high dose atorvastatin. Puccetti et al. (2010) conducted a case–control study to explore specific genetic and/or environmental factors to statin intolerance and they found a significant association between the C allele of rs4149056 SNP in the SLCO1B1 and myopathy in atorvastatin-treated patients (OR = 2.7, 95 % CI 1.3–4.9, *P* < 0.001). Since the genetic model under which the reported OR had been calculated was not available, we did not incorporate this study into our meta-analysis, which may influence our results.

The −388A>G is another common variant in the SLCO1B1 gene, which is in strong linkage disequilibrium with the −521T>C SNP (Link et al. 2008). Unlike rs4149056, rs2306283 SNP enhances liver uptake of pravastatin bringing about a reduced area under the curve for plasma pravastatin concentration (Niemi et al. 2004). The synthesis of studies on −388A>G polymorphism yielded a non-statistically significant protective effect on ADRs caused by any statin. But the results should be interpreted with caution because there were only two available studies incorporated to the meta-analyses. Therefore, more well-designed large sample size original studies are needed to identify the association.

When we performed the meta-analyses on association between SLCO1B1 −521T>C and ADRs caused by various statins, high heterogeneity was present in all genetic models except for the recessive model. The disparate definitions of ADRs, study design, characteristics of participants (i.e., age, sex, BMI, health status), statin type, dose and duration of treatment, and some other factors maybe the potential explanation. Unfortunately, we could only conduct the subgroup analysis for statin type but not other factors owing to the limited available data and small number of original studies. After stratification analyses, the *I*^2^ values were decreased to zero in the atorvastatin group but for the simvastatin the heterogeneity remained almost the same. In order to further explore the source of heterogeneity and to investigate the influence of individual study to the overall results, sensitivity analysis was performed. When we removed studies by de Keyser et al. (2014) respectively, the pooled effect estimates changed to be statistically significant in the heterozygote comparison. Both of the two studies reported insignificant reduced risk of dose decrease or switches in TC carriers, which may take place by chance, and might contributed more to the overall insignificant result. For simvastatin users, no significant association between −521T>C polymorphism and ADRs was found with the exclusion of one study by Carr et al. (2013) for the homozygote contrast. Possible explanation was that in this study, borderline statistically significant differences between cases and controls in terms of previous history of type 2 diabetes (*P* = 0.046), asthma (*P* = 0.080), and hypertension (*P* = 0.087) were determined, which might be confounders making the calculated OR biased consequently influencing the pooled result.

Several limitations in our study may affect the results. Firstly, it is noteworthy that the definitions of statin-induced ADRs varied among individual studies, which contributed to the presence of heterogeneity to a large extent. Further studies using uniform definitions are required to reach more definitive conclusions. Secondly, the crude and adjusted effect estimates were combined together due to the limited available data from the original studies. Moreover, the adjusting factors for each effect estimate were not completely consistent. As such, some potential confounding risk factors may be introduced to influence our pooled results, such as the differences in statin therapy including dose and duration of treatment. It is indicated that methodological improvement in the individual studies should be focused. Thirdly, the data from original studies were insufficient. We could not perform further stratification analyses to explore dose-, gender-, age-, BMI- and comedication-gene interactions. Additionally, the limited number of studies on −388A>G polymorphism lowered our power and the results of meta-analyses should be interpreted with caution. Fourthly, we only included articles published in English in four databases, relevant articles published in other databases and in other languages and unpublished studies may have been missed. What’s more, as the number of included studies was small, the power of detecting the publication bias in Begg’s and Egger’s test was low. Finally, the original studies were entirely or predominantly based on Caucasians, thus additional researches in other ethnicities are needed to generalize the findings.

In conclusion, our meta-analysis suggests that SLCO1B1 −521T>C polymorphism may be significantly associated with increased risk of statin-induced adverse reactions, especially in the simvastatin therapy. Conversely, the −521C minor allele might not modify the risk of atorvastatin-associated adverse effects. Besides, there may be no significant association between −388A>G polymorphism and statin-related adverse reactions. However, the finding should be interpreted with caution because of the small number of studies and small sample sizes. Well-designed epidemiological studies with large sample size in the treatment of various statins among different ethnicities should be carried out to confirm these associations and interaction between dose-, sex-, BMI-, extensive physical exercise-, comedication-gene should also be further investigated.

## References

[CR1] Baigent C, Keech A, Kearney PM, Blackwell L, Buck G, Pollicino C, Kirby A, Sourjina T, Peto R, Collins R, Simes R (2005). Efficacy and safety of cholesterol-lowering treatment: prospective meta-analysis of data from 90, 056 participants in 14 randomised trials of statins. Lancet.

[CR2] Begg CB, Mazumdar M (1994). Operating characteristics of a rank correlation test for publication bias. Biometrics.

[CR3] Brunham LR, Lansberg PJ, Zhang L, Miao F, Carter C, Hovingh GK, Visscher H, Jukema JW, Stalenhoef AF, Ross CJ, Carleton BC, Kastelein JJ, Hayden MR (2012). Differential effect of the rs4149056 variant in SLCO1B1 on myopathy associated with simvastatin and atorvastatin. Pharmacogenomics J.

[CR4] Carr DF, O’Meara H, Jorgensen AL, Campbell J, Hobbs M, McCann G, van Staa T, Pirmohamed M (2013). SLCO1B1 genetic variant associated with statin-induced myopathy: a proof-of-concept study using the clinical practice research datalink. Clin Pharmacol Ther.

[CR5] Chen C, Stock JL, Liu X, Shi J, Van Deusen JW, DiMattia DA, Dullea RG, de Morais SM (2008). Utility of a novel Oatp1b2 knockout mouse model for evaluating the role of Oatp1b2 in the hepatic uptake of model compounds. Drug Metab Dispos.

[CR6] Cota GF, de Sousa MR, Fereguetti TO, Rabello A (2013). Efficacy of anti-leishmania therapy in visceral leishmaniasis among HIV infected patients: a systematic review with indirect comparison. PLoS Negl Trop Dis.

[CR7] Crowther M, Lim W, Crowther MA (2010). Systematic review and meta-analysis methodology. Blood.

[CR8] Danik JS, MacFadyen J, Nyberg F, Ridker P (2012). Lack of association between polymorphisms in the SLC01B1 gene and clinical myalgia following rosuvastatin therapy. J Am Coll Cardiol.

[CR9] Danik JS, Chasman DI, MacFadyen JG, Nyberg F, Barratt BJ, Ridker PM (2013). Lack of association between SLCO1B1 polymorphisms and clinical myalgia following rosuvastatin therapy. Am Heart J.

[CR10] de Keyser CE, Becker ML, Maitland-Van Der Zee A-H, Uitterlinden AG, Hofman A, Visser LE, Stricker BH (2012). The SLCO1B1 C.521T>C polymorphism and the risk of adverse reactions during simvastatin and atorvastatin therapy. Pharmacoepidemiol Drug Saf.

[CR11] de Keyser CE, Stricker BH, Becker ML (2012). Research highlights. Pharmacogenomics.

[CR12] de Keyser CE, Peters BJ, Becker ML, Visser LE, Uitterlinden AG, Klungel OH, Verstuyft C, Hofman A, Maitland-van der Zee AH, Stricker BH (2014). The SLCO1B1 c.521T>C polymorphism is associated with dose decrease or switching during statin therapy in the Rotterdam Study. Pharmacogenet Genomics.

[CR13] de Lemos JA, Blazing MA, Wiviott SD, Lewis EF, Fox KA, White HD, Rouleau JL, Pedersen TR, Gardner LH, Mukherjee R, Ramsey KE, Palmisano J, Bilheimer DW, Pfeffer MA, Califf RM, Braunwald E (2004). Early intensive vs a delayed conservative simvastatin strategy in patients with acute coronary syndromes: phase Z of the A to Z trial. JAMA.

[CR14] Dendramis G (2011). Interindividual differences in the response to statin therapy and gene polymorphisms related to myopathy during statin therapy. G Ital Cardiol (Rome).

[CR15] DerSimonian R, Laird N (1986). Meta-analysis in clinical trials. Control Clin Trials.

[CR16] Dlouha D, Hubacek J, Adamkova V, Snejderlova M, Ceska R, Vrablik M (2013). Association between common variants in the SLCO1B1 gene and statin-induced myopathy. Cardiology (Switzerland).

[CR17] Dmitry S, Grigory S, Andrey G, Tatyana B (2013). The frequency of allele genotypes of gene SLCO1B1null5 in Russian patients suffering from hyperlipidemia. Drug Metabol Drug Interact.

[CR18] Dolgin E (2013). Pharmacogenetic tests yield bonus benefit: better drug adherence. Nat Med.

[CR19] Donnelly LA, Doney AS, Tavendale R, Lang CC, Pearson ER, Colhoun HM, McCarthy MI, Hattersley AT, Morris AD, Palmer CN (2011). Common nonsynonymous substitutions in SLCO1B1 predispose to statin intolerance in routinely treated individuals with type 2 diabetes: a go-DARTS study. Clin Pharmacol Ther.

[CR20] Egger M, Davey Smith G, Schneider M, Minder C (1997). Bias in meta-analysis detected by a simple, graphical test. BMJ.

[CR21] Feng Q, Wilke RA, Baye TM (2012). Individualized risk for statin-induced myopathy: current knowledge, emerging challenges and potential solutions. Pharmacogenomics.

[CR22] Fiegenbaum M, da Silveira FR, Van der Sand CR, Van der Sand LC, Ferreira ME, Pires RC, Hutz MH (2005). The role of common variants of ABCB1, CYP3A4, and CYP3A5 genes in lipid-lowering efficacy and safety of simvastatin treatment. Clin Pharmacol Ther.

[CR23] Frudakis TN, Thomas MJ, Ginjupalli SN, Handelin B, Gabriel R, Gomez HJ (2007). CYP2D6*4 polymorphism is associated with statin-induced muscle effects. Pharmacogenet Genomics.

[CR24] Furihata T, Satoh N, Ohishi T, Ugajin M, Kameyama Y, Morimoto K, Matsumoto S, Yamashita K, Kobayashi K, Chiba K (2009). Functional analysis of a mutation in the SLCO1B1 gene (c.1628T>G) identified in a Japanese patient with pravastatin-induced myopathy. Pharmacogenomics J.

[CR25] Ghatak A, Faheem O, Thompson PD (2010). The genetics of statin-induced myopathy. Atherosclerosis.

[CR26] Graham DJ, Staffa JA, Shatin D, Andrade SE, Schech SD, La Grenade L, Gurwitz JH, Chan KA, Goodman MJ, Platt R (2004). Incidence of hospitalized rhabdomyolysis in patients treated with lipid-lowering drugs. JAMA.

[CR27] Higgins JP, Thompson SG (2002). Quantifying heterogeneity in a meta-analysis. Stat Med.

[CR28] Ho PM, Magid DJ, Shetterly SM, Olson KL, Maddox TM, Peterson PN, Masoudi FA, Rumsfeld JS (2008). Medication nonadherence is associated with a broad range of adverse outcomes in patients with coronary artery disease. Am Heart J.

[CR29] Hopewell JC, Offer A, Parish S, Haynes R, Li J, Jiang L, Lathrop M, Armitage J, Collins R (2012). Environmental and genetic risk factors for myopathy in Chinese participants from HPS2-THRIVE. Eur Heart J.

[CR30] Ishikawa C, Ozaki H, Nakajima T, Ishii T, Kanai S, Anjo S, Shirai K, Inoue I (2004). A frameshift variant of CYP2C8 was identified in a patient who suffered from rhabdomyolysis after administration of cerivastatin. J Hum Genet.

[CR31] Iwai M, Suzuki H, Ieiri I, Otsubo K, Sugiyama Y (2004). Functional analysis of single nucleotide polymorphisms of hepatic organic anion transporter OATP1B1 (OATP-C). Pharmacogenetics.

[CR32] Johansen C, Phillips MS, Dube M-P, Wang J, Lin T, Ban MR, Kennedy BA, Tardif J-C, Hegele RA (2010). SLCO1B1 is not associated with statin-induced myopathy in patients from a tertiary referral lipid clinic. Arterioscler Thromb Vasc Biol.

[CR33] Kamatani N, Mushiroda T (2011). Use of the data from genome-wide association study (GWAS) for pharmacogenomics and new drug development: focusing on transporter genes. J Pharmacol Sci.

[CR34] Kameyama Y, Yamashita K, Kobayashi K, Hosokawa M, Chiba K (2005). Functional characterization of SLCO1B1 (OATP-C) variants, SLCO1B1*5, SLCO1B1*15 and SLCO1B1*15 + C1007G, by using transient expression systems of HeLa and HEK293 cells. Pharmacogenet Genomics.

[CR35] Khan AA, Ding K, Khader S, Kullo IJ (2011). Association of a polymorphism in SLCO1B1 with statin-induced myalgias, myositis and myopathy: an electronic medical record based pharmacogenetic study. J Am Coll Cardiol.

[CR36] König J, Seithel A, Gradhand U, Fromm MF (2006). Pharmacogenomics of human OATP transporters. Naunyn Schmiedebergs Arch Pharmacol.

[CR37] Kroemer HK (2010). Cardiovascular drug transport: influence of genetics and disease. Drug Metab Rev.

[CR38] Linde R, Peng L, Desai M, Feldman D (2010). The role of vitamin D and SLCO1B1*5 gene polymorphism in statin-associated myalgias. Dermatoendocrinol.

[CR39] Link E, Parish S, Armitage J, Bowman L, Heath S, Matsuda F, Gut I, Lathrop M, Collins R (2008). SLCO1B1 variants and statin-induced myopathy—a genomewide study. N Engl J Med.

[CR40] Mafalda M, Leticia B, Claudia CB, Luisa M-V (2013). Characterization of the functional genetic variants relevant to statin response in the Azores Islands population (Portugal). Drug Metabol Drug Interact.

[CR41] Mantel N, Haenszel W (1959). Statistical aspects of the analysis of data from retrospective studies of disease. J Natl Cancer Inst.

[CR42] Marciante KD, Durda JP, Heckbert SR, Lumley T, Rice K, McKnight B, Totah RA, Tamraz B, Kroetz DL, Fukushima H, Kaspera R, Bis JC, Glazer NL, Li G, Austin TR, Taylor KD, Rotter JI, Jaquish CE, Kwok PY, Tracy RP, Psaty BM (2011). Cerivastatin, genetic variants, and the risk of rhabdomyolysis. Pharmacogenet Genomics.

[CR43] McClure DL, Valuck RJ, Glanz M, Murphy JR, Hokanson JE (2007). Statin and statin-fibrate use was significantly associated with increased myositis risk in a managed care population. J Clin Epidemiol.

[CR44] Meador BM, Huey KA (2010). Statin-associated myopathy and its exacerbation with exercise. Muscle Nerve.

[CR45] Nakamura Y (2008). Pharmacogenomics and drug toxicity. N Engl J Med.

[CR46] Niemi M, Schaeffeler E, Lang T, Fromm MF, Neuvonen M, Kyrklund C, Backman JT, Kerb R, Schwab M, Neuvonen PJ, Eichelbaum M, Kivisto KT (2004). High plasma pravastatin concentrations are associated with single nucleotide polymorphisms and haplotypes of organic anion transporting polypeptide-C (OATP-C, SLCO1B1). Pharmacogenetics.

[CR47] Niemi M, Pasanen MK, Neuvonen PJ (2011). Organic anion transporting polypeptide 1B1: a genetically polymorphic transporter of major importance for hepatic drug uptake. Pharmacol Rev.

[CR48] Nozawa T, Nakajima M, Tamai I, Noda K, Nezu J, Sai Y, Tsuji A, Yokoi T (2002). Genetic polymorphisms of human organic anion transporters OATP-C (SLC21A6) and OATP-B (SLC21A9): allele frequencies in the Japanese population and functional analysis. J Pharmacol Exp Ther.

[CR49] Oh J, Ban MR, Miskie BA, Pollex RL, Hegele RA (2007). Genetic determinants of statin intolerance. Lipids Health Dis.

[CR50] Pasanen MK, Neuvonen M, Neuvonen PJ, Niemi M (2006). SLCO1B1 polymorphism markedly affects the pharmacokinetics of simvastatin acid. Pharmacogenet Genomics.

[CR51] Pasanen MK, Fredrikson H, Neuvonen PJ, Niemi M (2007). Different effects of SLCO1B1 polymorphism on the pharmacokinetics of atorvastatin and rosuvastatin. Clin Pharmacol Ther.

[CR52] Patel J, Abd T, Blumenthal RS, Nasir K, Superko HR (2014). Genetics and personalized medicine–a role in statin therapy?. Curr Atheroscler Rep.

[CR53] Petrov VI, Smuseva ON, Solovkina YV (2013). Integrated assessment of statin-associated muscle damage predictors in patients with ischemic heart disease. Ration Pharmacother Cardiol.

[CR54] Pranculis A, Kucinskas V (2013). Pharmacogenomic landscape of the Lithuanian population. Drug Metabol Drug Interact.

[CR55] Puccetti L, Ciani F, Auteri A (2010). Genetic involvement in statins induced myopathy. Preliminary data from an observational case-control study. Atherosclerosis.

[CR56] Rodriguez S, Gaunt TR, Day IN (2009). Hardy-Weinberg equilibrium testing of biological ascertainment for Mendelian randomization studies. Am J Epidemiol.

[CR57] Santos PC, Gagliardi AC, Miname MH, Chacra AP, Santos RD, Krieger JE, Pereira AC (2012). SLCO1B1 haplotypes are not associated with atorvastatin-induced myalgia in Brazilian patients with familial hypercholesterolemia. Eur J Clin Pharmacol.

[CR58] Schech S, Graham D, Staffa J, Andrade SE, La Grenade L, Burgess M, Blough D, Stergachis A, Chan KA, Platt R, Shatin D (2007). Risk factors for statin-associated rhabdomyolysis. Pharmacoepidemiol Drug Saf.

[CR59] Simons LA, Levis G, Simons J (1996). Apparent discontinuation rates in patients prescribed lipid-lowering drugs. Med J Aust.

[CR60] Stewart A (2013) SLCO1B1 Polymorphisms and statin-induced myopathy. PLoS Curr 5 10.1371/currents.eogt.d21e7f0c58463571bb0d9d3a19b82203 PMC3871416 24459608

[CR61] Sychev DA, Shuev GN, Prokofiev AB (2013). Applied aspects of SLCO1B1 pharmacogenetic testing for predicting of statin-induced myopathy and personalization of statins therapy. Ration Pharmacother Cardiol.

[CR62] Tamraz B, Fukushima H, Wolfe AR, Kaspera R, Totah RA, Floyd JS, Ma B, Chu C, Marciante KD, Heckbert SR, Psaty BM, Kroetz DL, Kwok PY (2013). OATP1B1-related drug-drug and drug-gene interactions as potential risk factors for cerivastatin-induced rhabdomyolysis. Pharmacogenet Genomics.

[CR63] Thompson PD, Clarkson PM, Rosenson RS (2006). An assessment of statin safety by muscle experts. Am J Cardiol.

[CR64] Tirona RG, Leake BF, Merino G, Kim RB (2001). Polymorphisms in OATP-C: identification of multiple allelic variants associated with altered transport activity among European- and African-Americans. J Biol Chem.

[CR65] Toms TE, Smith JP, Panoulas VF, Douglas KM, Saratzis AN, Kitas GD (2009). Prevalence of risk factors for statin-induced myopathy in rheumatoid arthritis patients: does the SLCO1B1 gene play a role?. Rheumatology (Oxford).

[CR66] Toms TE, Smith JP, Panoulas VF, Douglas KM, Saratzis AN, Kitas GD (2010). Prevalence of risk factors for statin-induced myopathy in rheumatoid arthritis patients. Musculoskeletal Care.

[CR67] Vladutiu GD (2008). Genetic predisposition to statin myopathy. Curr Opin Rheumatol.

[CR68] Vladutiu GD, Simmons Z, Isackson PJ, Tarnopolsky M, Peltier WL, Barboi AC, Sripathi N, Wortmann RL, Phillips PS (2006). Genetic risk factors associated with lipid-lowering drug-induced myopathies. Muscle Nerve.

[CR69] Voora D, Ali S, Shah SH, Reed CR, Salisbury B, Ginsburg GS (2008) Association of statin-induced musculoskeletal side effects with sex and a hepatic uptake transporter reduced function Allele, SLC01B1*5. Circulation 118:S326

[CR70] Voora D, Shah SH, Spasojevic I, Ali S, Reed CR, Salisbury BA, Ginsburg GS (2009). The SLCO1B1*5 genetic variant is associated with statin-induced side effects. J Am Coll Cardiol.

[CR71] Wilke RA, Moore JH, Burmester JK (2005). Relative impact of CYP3A genotype and concomitant medication on the severity of atorvastatin-induced muscle damage. Pharmacogenet Genomics.

[CR72] Wilke RA, Reif DM, Moore JH (2005). Combinatorial pharmacogenetics. Nat Rev Drug Discov.

[CR73] Wilke RA, Ramsey LB, Johnson SG, Maxwell WD, McLeod HL, Voora D, Krauss RM, Roden DM, Feng Q, Cooper-Dehoff RM, Gong L, Klein TE, Wadelius M, Niemi M (2012). The clinical pharmacogenomics implementation consortium: CPIC guideline for SLCO1B1 and simvastatin-induced myopathy. Clin Pharmacol Ther.

[CR74] Wright JL, Zhou S, Preobrazhenska O, Marshall C, Sin DD, Laher I, Golbidi S, Churg AM (2011). Statin reverses smoke-induced pulmonary hypertension and prevents emphysema but not airway remodeling. Am J Respir Crit Care Med.

